# Fitness of Nutrition Regulation in a Caterpillar Pest *Mythimna separata* (Walker): Insights from the Geometric Framework

**DOI:** 10.3390/insects14120937

**Published:** 2023-12-08

**Authors:** Shaolei Sun, Zhen Yang, Jinchan Ren, Tongxian Liu, Xiangfeng Jing

**Affiliations:** 1 Key Laboratory of Integrated Pest Management on the Loess Plateau of Ministry of Agriculture, College of Plant Protection, Northwest A&F University, Xianyang 712100, China; ssl_0207@163.com (S.S.); yangzhen19930518@163.com (Z.Y.); rjc@nwafu.edu.cn (J.R.); tx.liu@gzu.edu.cn (T.L.); 2 Fishery College, Zhejiang Ocean University, Zhoushan 316022, China

**Keywords:** fitness landscape, proteins, carbohydrates, nutrient regulation, *Mythimna separata*

## Abstract

**Simple Summary:**

A critical environmental factor that affects the growth and development of insect herbivores is food quality, and insect herbivores require the correct blend and balance of different nutrients in order to meet their physiological demands. For the first time, we investigated the nutritional regulation of the oriental armyworm, *Mythimna separata* (walker), and the association between insect performance (i.e., developmental time, insect mass, and reproductive response) and nutrients. Caterpillars had different intake targets for two macronutrients, proteins and carbohydrates, at two larval stages, and food consumption and nutrient intake were closely correlated with the content of protein and carbohydrate, respectively. Interestingly, we also found that the trade-off between nutritional regulation and fecundity, a phenomenon often seen in this migrating species, may be attributed to food quality and, subsequently, physiological preparation. These results help us better understand the behavior of this economically important pest in the field.

**Abstract:**

In nature, plants can contain variable nutrients depending upon the species, tissue, and developmental stage. Insect herbivores may regulate their nutrient intake behaviorally and physio- logically when encountering different foods. This study examined the nutritional regulation of the oriental armyworm, *Mythimna separata*, for the first time. In one experiment, we allowed the cater-pillars to choose between two nutritionally balanced but complementary diets. The caterpillars did not randomly consume the paired foods, but instead chose between the nutritionally balanced but complementary diets. This intake behavior was found to change with their developmental stages. Furthermore, the nutrient concentrations in food significantly impacted the insect’s performance. In the other experiment, caterpillars were given one of eleven diets that reflected the different nutrient conditions in the field. The results showed that proteins were significantly associated with developmental time and fecundity. For example, by consuming protein-biased food, the caterpillars developed faster and produced more eggs. In contrast, carbohydrates were more strongly linked to lipid accumulation, and caterpillars accumulated more lipids when consuming the carbohydrate-biased food. Moreover, the caterpillars were also found to actively regulate their intake of proteins and carbohydrates based on food quality and to physiologically prepare for subsequent life stages. These findings enhance our understanding of how *M. separata* feeds and responds to different nutritional environments in the field, which could have implications for managing insect herbivores in agricultural settings.

## 1. Introduction

Insects have evolved various strategies to cope with the variable nutrient composition of their plant hosts. They exhibit feeding preferences for plants or plant tissues that provide the necessary nutrients in the right proportions [1,2]. For example, some insects have a preference for protein-rich tissues, while others prefer carbohydrate-rich tissues [3]. Inadequate nutrient intake can lead to reduced growth rates, delayed development, and decreased reproductive success [4]. On the other hand, an excess of certain nutrients may have negative effects on insect fitness [5]. In addition to feeding preferences, insects also have physiological adaptations to optimize nutrient acquisition [6,7]. Overall, the ability of insect herbivores to obtain the correct blend and balance of nutrients from their plant hosts is crucial for their survival and successful completion of their life cycle [8].

The nutritional status of insects can greatly influence their growth and development. Previous research has mostly focused on the effect of a single nutrient, but often overlooks the complexity of the nutrient matrix in food, that is, the individual and mutual influence of multiple nutrients [9,10]. The development of the Geometric Framework for nutrition provides a powerful tool to study this issue in a multidimensional and variable nutritional environment [11]. Using this tool, the adaptive responses of some insects to proteins and carbohydrates have been studied [12,13,14,15,16,17,18,19,20,21,22,23]. Two characteristics, larval development period and pupal mass, are often used to determine the adaptability of insects, but the association between reproductive response and nutrient intake is rarely studied [19,20,21]. To our knowledge, only one study has ever measured the egg production of a caterpillar, *Heliothis virescens*, on foods with different protein/carbohydrate contents [19]. In addition, the impact of specific nutrients on the fecundity of fruit flies, *Drosophila melanogaster*, was also studied [24,25,26]. However, these studies on fruit flies mainly focused on the impact of dietary consumption during the adult stage. However, larval nutrition can play a key role in shaping fecundity, especially for lepidopteran insects [27,28,29,30,31,32,33,34]. 

The oriental armyworm, *Mythimna separata*, is a major lepidopteran pest of graminaceous crops [35,36,37,38], and the nutritional regulation of this important pest has not been investigated. As a typical migrating animal, this insect performs poleward migration from lower-latitude winter habitats to exploit new resources in temporary areas where they cannot survive over winter. In this study, we investigated whether *M. separata* can regulate its nutrient intake, through the use of artificial diets which mimicked its nutrient environment in the field, and how the larval nutritional status affected insect performance, especially fecundity. We hypothesized that *M. separata* can reach a nutritional target when allowed to choose between protein-biased foods and carbohydrate-biased foods, and the nutrient composition can significantly affect fecundity. To test this hypothesis, we designed two experiments in this study. The first experiment was a choice experiment, and the caterpillars were individually allowed to choose between the pairing diets that had different protein and carbohydrate contents. Through this, we determined how the caterpillars regulate their nutrient intake to meet their nutritional target. In the second experiment, the caterpillars were individually restricted to one of eleven diets to evaluate the insect’s behavioral and physiological responses. The results help us to understand how these insects regulate their nutrient intake and prepare themselves physiologically for their typical marching and migration behaviors. 

## 2. Materials and Methods

### 2.1. Insect Rearing and Experimental Chambers

The study utilized the oriental armyworms, *M. separata*, which were procured from Baiyun Industry Co., Ltd. in Jiyuan, China. The insects were kept in plastic containers measuring 9 cm by 6 cm. The larvae were fed two nutritionally complementary but imbalanced foods (p28:c14 vs. p14:c28) so that the insects have similar nutritional states when self-selecting either of the two foods. The developmental stage of the insects was recorded twice daily. When a larva molted to the 5th instar stage, it was weighed to record its initial fresh mass and then transferred to a plastic Petri dish with a diameter of 9 cm. The top lid of each Petri dish had five ventilation holes, each approximately 1 mm in diameter. Each Petri dish contained either two food blocks for the choice experiment or one food block for the no-choice experiment. All procedures were carried out in an incubator maintained at a temperature of 25 ± 1 °C with a 14:10 h light/dark photoperiod.

### 2.2. Experimental Diets 

**Figure 1:**
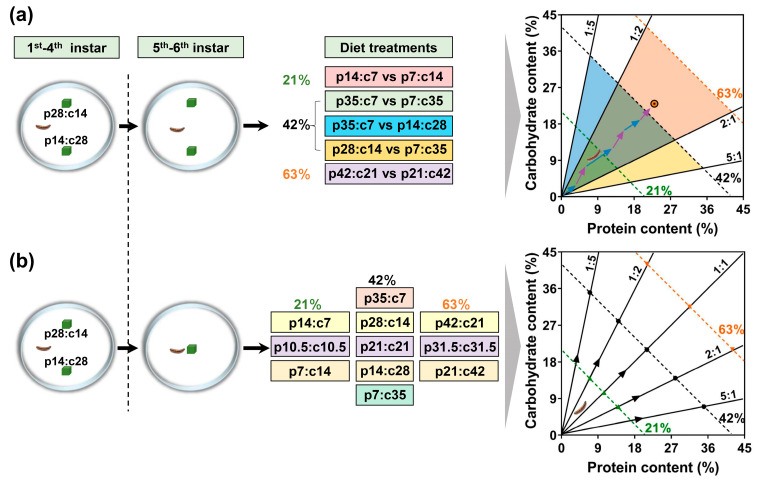
Experimental design of choice treatments (**a**) and no-choice treatments (**b**). Each caterpillar was fed a pair of nutritionally complementary foods (p28:c14 vs. p14:c28) from the 1st instar to the 4th instar in a plastic container so all experimental animals had similar nutritional statuses. The newly molted 5th instar caterpillars were used in the following experiments. In the choice treatments (**a**), each caterpillar was given one of five food pairings (p14:c7 vs. p7:c14, p35:c7 vs. p7:c35, p35:c7 vs. p14:c28, p28:c14 vs. p7:c35, or p42:c21 vs. p21:c42 under different colored blocks). The first pairing had the different ratio of protein to carbohydrate under 21% concentration of these nutrients. The pairings of p35:c7 vs. p7:c35, p35:c7 vs. p14:c28, and p28:c14 vs. p7:c35 had the same total concentration (42%) but different ratios of protein to carbohydrate. The last pairing had the different ratio of protein to carbohydrate under 63% concentrations of these nutrients. The different colored arrows represent the self-selecting either of the two foods under choice treatments. In the no-choice treatments (**b**), each caterpillar was given one of eleven foods. These eleven foods under different colored blocks can be divided into 3 groups according to the total concentrations of the nutrients, i.e., 21%, 42%, and 63%. In each group, diets differed in the ratio of protein to carbohydrate. The ratios are represented by the rails projecting from the origin in the coordination space, and the nutrient concentrations are represented by the dotted lines.

A diet developed for *M. separata* was used in this experiment [39]. In this diet, corn leaf powder accounted for 26% of the diet’s dry mass. The amounts of proteins and carbohydrates in corn leaf powder were measured. A mixture of casein, peptone, and albumen (ratio = 3:1:1) as the protein source and sucrose as the digestible carbohydrate source were used to adjust the composition of proteins and carbohydrates. The other chemical components of these diets were identical. In total, 11 artificial diets that varied in protein (p) and digestible carbohydrate (c) content were prepared (Figure 1), including p14:c7, p10.5:c10.5, p7:c14, p35:c7, p28:c14, p21:c21, p14:c28, p7:c35, p42:c21, p31.5:c31.5, and p21:c42. The sum of the two nutrients accounted for 21% of the dry mass for the first three diets, and 63% for the last three. The other diets contained 42% of these two nutrients. At each concentration, the diet varied from protein-biased to carbohydrate-biased. The nutritional range of proteins and carbohydrates reflected the concentrations in corn plants [40].

### 2.3. Experimental Treatments

Two independent experiments were performed. In the choice treatment, each caterpillar was assigned at random to one of the five food pairings: (1) p14:c7 vs. p7:c14, (2) p35:c7 vs. p7:c35, (3) p35:c7 vs. p14:c28, (4) p28:c14 vs. p7:c35, or (5) p42:c21 vs. p21:c42. The distance between the two food blocks was approximately three times the length of the newly molted larvae, e.g., 6 cm for 5th instar larvae and 9 cm for 6th instar larvae [41]. Twenty replicates were set up for each treatment (Figure 1a). In the no-choice experiment, each caterpillar was randomly assigned to 1 of the 11 diets. These diets reflected a nutritional (protein and carbohydrate) landscape which the caterpillar may encounter in the field (Figure 1b). Thirty replicates were set up for each treatment.

### 2.4. Experimental Protocol

Each caterpillar was transferred into a Petri dish, which was sealed by parafilm and kept under the experimental conditions. Food blocks were weighed and then fed to the caterpillars. Five control blocks were set in an empty Petri dish to construct a regression equation to calculate the initial dry mass of the food blocks fed to the caterpillars in each treatment [42]. The food blocks were replaced every two days until the larvae ceased to feed (a sign of pupation). The remaining diet was collected and dried to a constant mass at 50 °C. Food consumption for each caterpillar was calculated as the dry mass difference between the initial food and the remaining food. Each pupa was weighed two days after pupation. All pupae were sexed, and 10 pupae (5 female and 5 male) for each treatment were randomly selected and allowed to eclose. After eclosion, one female and one male were paired in a plastic container (9 cm × 12 cm), and a 10% sucrose solution was provided via a cotton wick using a 10 mL centrifugal tube. Paper towel strips (9 cm × 1 cm) hung from the top were provided for females to lay eggs. The paper towel strips were collected every day for 10 days and the eggs were counted. Other pupae were frozen at −20 °C. These pupae were freeze-dried and their lipid content was measured by three rounds of chloroform extractions as described previously [43]. Thirty newly molted 5th instar larvae were used to determine the initial lipid content. Lipid accumulation was determined as the lipid content difference between the newly molted 5th instar larvae and the pupae.

### 2.5. Statistical Analysis

In the choice experiment, paired *t* tests were used to compare the consumption of the two foods in each treatment, and ANOVA was used for the comparison of food consumption between different treatments. Multiple analysis of covariance (MANCOVA) was used for the comparison of nutrient consumption between different treatments for the 5th instar and the 6th instar larvae separately, using the treatment (food pairing) as the main effect in the model and the initial 5th instar larval mass as a covariate [44,45]. A Kaplan–Meier survival analysis (specifically the Mantel Cox test) was used to determine differences in developmental time between different treatments. Pupal mass was analyzed using analysis of covariate (ANCOVA), with the initial 5th instar larval mass as a covariate. Egg production was compared between different treatments using ANOVA. Lipid accumulation was analyzed using ANCOVA, and carbohydrate consumption was used as a covariate. Blom’s method was used to obtain normal distributions when necessary. All analyses were conducted in Statistical Product and Service Solutions V.25.0 software (SPSS, Chicago, IL, USA), and figures were generated using GraphPad Prism 8.00.

In the no-choice experiment, we used the response-surface methodology to estimate and visualize how food consumption and nutrient intake, development time, pupal mass, fecundity, and lipid accumulation changed with varying concentrations and ratios of proteins and carbohydrates in the 5th instar and the two instar stages, respectively [17,46]. The full models and the corresponding response surface figures were generated with a central-composite design from Design-Expert V.12.

## 3. Results

### 3.1. Choice Experiment

#### 3.1.1. Food and Nutrients Consumed

**Figure 2:**
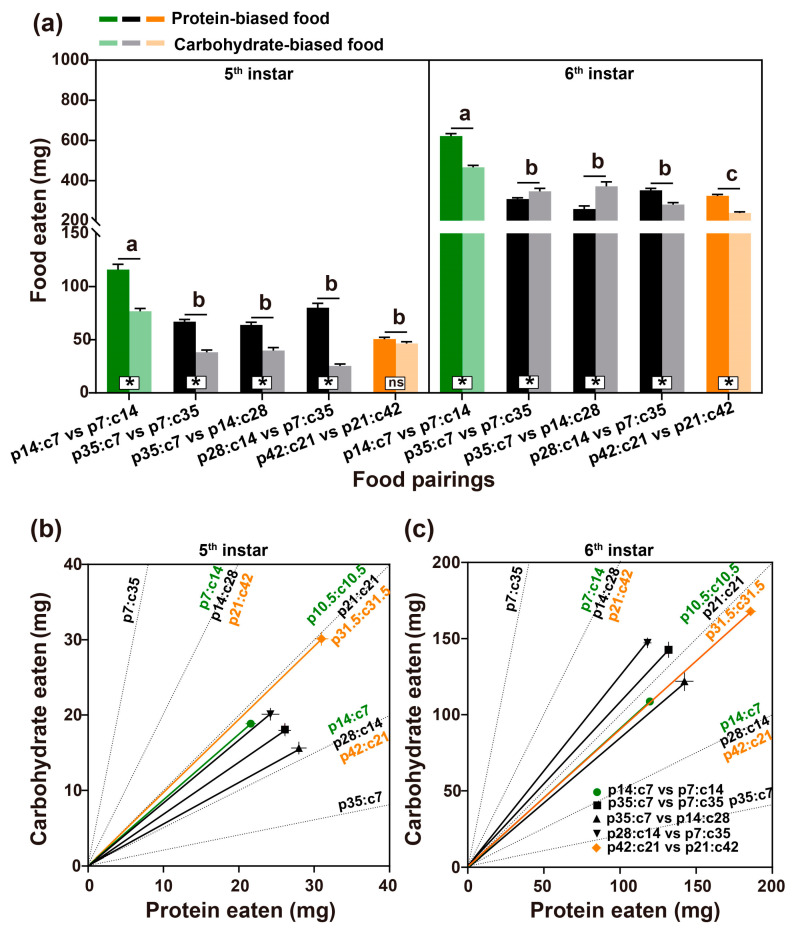
(**a**) Diet consumption (mean ± SE) of the caterpillars in the choice experiment. Different colors represent the different nutrient concentrations. Green represents the 21% food pairing, black represents the 42% food pairings, and orange represents the 63% food pairing. For each pairing, the bar with a dark color represents protein-biased food, and the light color bar represents carbohydrate-biased food. (**b**,**c**) The cumulative intake (bivariate mean ± SE) of proteins and carbohydrates over the 5th instar and 6th instar stages. The green symbol represents the 21% food pairing, black symbols represent the 42% food pairings, and the orange symbol represents the 63% food pairing. The dotted lines radiating from the origin represented five nutrient ratios of the food treatments from the highest p/c ratio to the lowest ratio. The line connecting the origin and filled circle symbol represents the intake trajectory. * indicates the significant difference, and ns represents nonsignificance. The total amount of food eaten on the five treatments were compared with the lower case letters, e.g., a, b, c, representing the significant difference between treatments.

The *M. separata* caterpillars showed a clear preference for a protein-biased diet during the 5th instar stage except for the pair with the highest nutrient concentration (Figure 2a; p14:c7 vs. p7:c14: t_19_ = 6.29, *p* < 0.001; p35:c7 vs. p7:c35: t_19_ = 16.35, *p* < 0.001; p35:c7 vs. p14:c28: t_19_ = 6.99, *p* < 0.001; p28:c14 vs. p7:c35: t_19_ = 11.47, *p* < 0.001; p42:c21 vs. p21:c42: t_19_ = 1.84, *p* = 0.081). The larval feeding pattern was altered for the larvae fed the middle concentration (42%) during the 6th instar stage. The consumption of the carbohydrate-biased food exceeded the protein-biased food for the pairs of p35:c7 vs. p7:c35 and p35:c7 vs. p14:c28 (p35:c7 vs. p7:c35: t_19_ = −2.24, *p* = 0.038; p35:c7 vs. p14:c28: t_19_ = −3.98, *p* = 0.001). For the pair of p28:c14 vs. p7:c35, the larvae still consumed significantly more of the protein-biased diet but the proportion of the carbohydrate-biased food consumption increased compared to the 5th instar insects.

Consistently, a multivariate analysis indicated there was a significant effect of food pairing on nutrient intake (Table 1). Thus, both the 5th instar and the 6th instar insects did not feed indiscriminately. However, nutrient intake was not tightly regulated. The self-selected p/c ratio was different across the five treatments for both 5th instar and 6th instar insects (Figure 2b; 5th instar: F_4,95_ = 51.53, *p* < 0.001; 6th instar: F_4,95_ = 27.41, *p* < 0.001). 

#### 3.1.2. Performance

**Figure 3:**
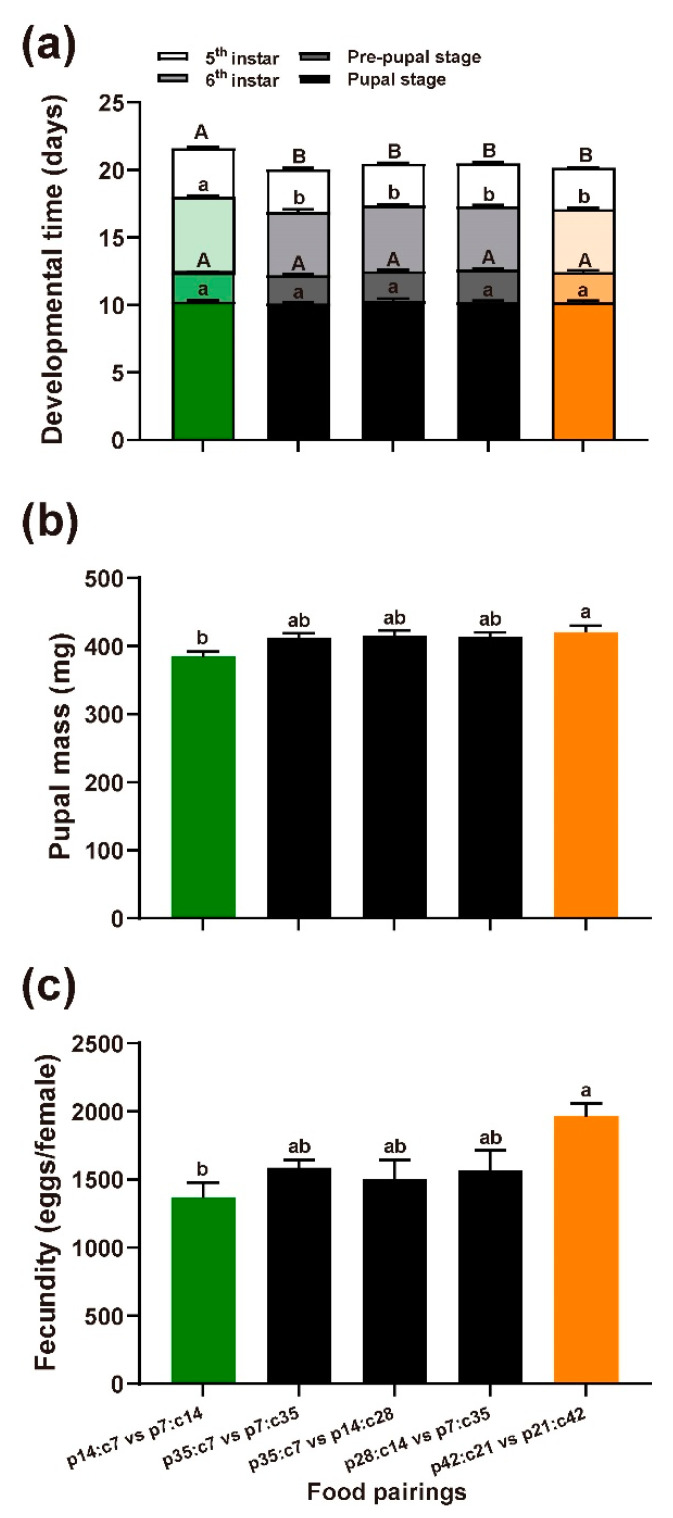
Insect performance in the choice treatments. Green color represents insect performance under the 21% food pairing, black represents insect performance under the 42% food pairings, and orange represents insect performance under the 63% food pairing. (**a**) Developmental time of four stages. Upper case letters represent the significant difference in the developmental time of the 5th instar and the pre-pupal stage between treatments. Lower case letters represent the significant difference in the developmental time of the 6th instar and the pupal stage between treatments; (**b**) pupal mass; (**c**) egg production. Means ± SE are presented.

In Figure 3a, the durations of the 5th and 6th caterpillar instar stages are depicted and show longer durations under the lowest nutrient concentration food pairings (5th instar: χ^2^ = 18.32, df = 4, *p* = 0.001; 6th instar: χ^2^ = 29.10, df = 4, *p* < 0.001). The food pairings, however, did not affect the durations of the prepupal and pupal stages (pre-pupal stage: χ^2^ = 6.12, df = 4, *p* = 0.191; pupal stage: χ^2^ = 3.00, df = 4, *p* = 0.558). It was observed that a higher nutrient concentration in the food pairing led to a heavier pupal mass (Figure 3b; F_4,94_ = 3.45, *p* = 0.011). The initial larval mass, however, did not significantly impact the pupal mass (F_1,94_ = 0.442, *p* = 0.724). Lastly, dietary nutrient concentration significantly impacted the fecundity, with a higher nutrient concentration leading to a greater production of eggs (Figure 3c; F_4,30_ = 3.64, *p* = 0.016).

#### 3.1.3. Lipid Accumulation

**Figure 4:**
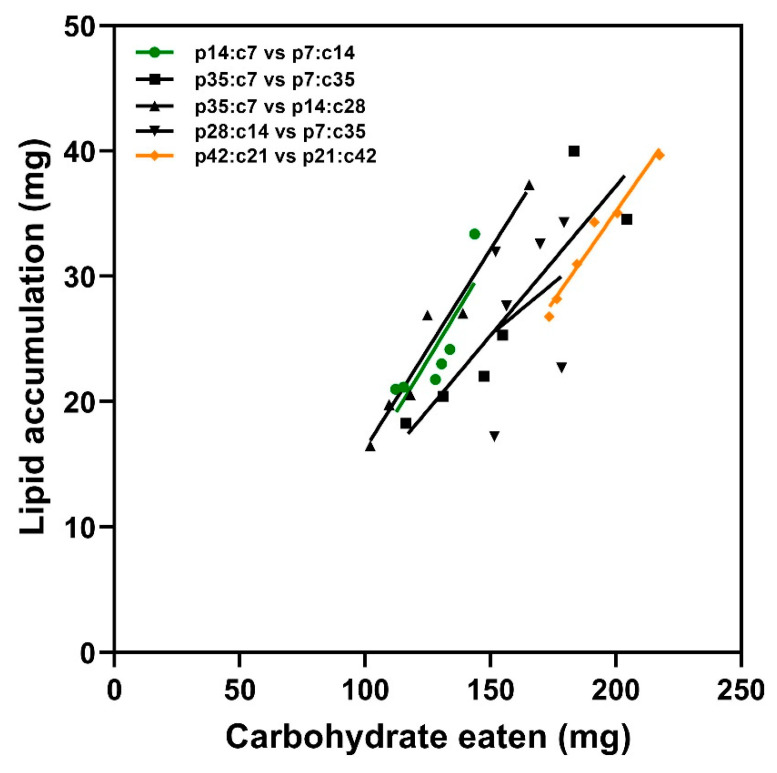
The conversion of ingested carbohydrates to body lipids in the choice experiment. Each point represents an individual insect that pupated, and simple linear regressions were fitted across the five choice treatments to describe the conversion efficiencies.

There was a noticeable impact of carbohydrate intake on lipid accumulation in *M. separata* caterpillars (Figure 4, F_1,24_ = 53.18, *p* < 0.001). Consistently, simple linear regressions between lipid accumulation and carbohydrate intake indicated that the caterpillars had similar efficiencies in converting carbohydrates into lipids regardless of the food pairings (F_4,20_ = 0.36, *p* = 0.832). However, no considerable influence of the food pairings on this association was observed (F_4,24_ = 2.70, *p* = 0.055). 

### 3.2. No-Choice Experiment

#### 3.2.1. Food and Nutrient Consumed across the Nutrient Landscape

**Figure 5:**
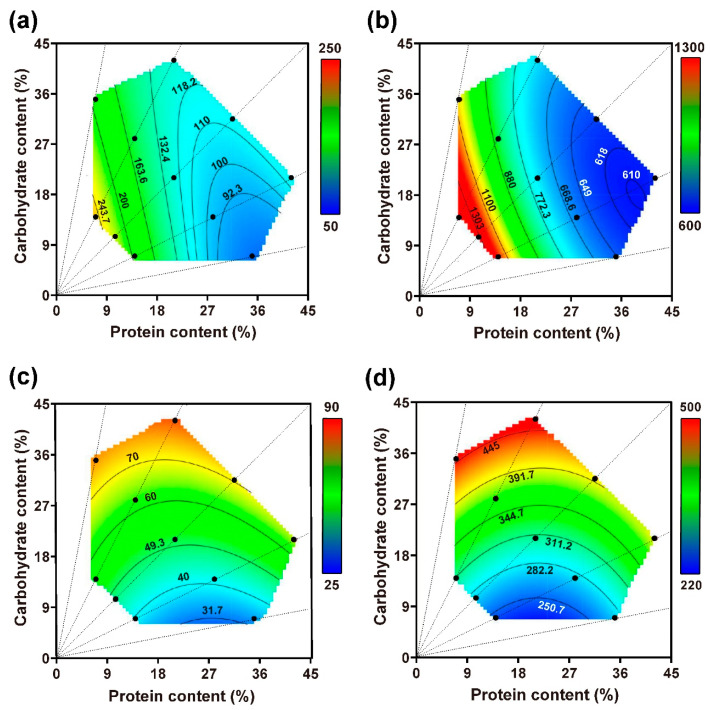
Thin-plate spline visualizations of the response surface for food consumption (**a**,**b**) and nutrient intake (**c**,**d**) across the nutrient landscape in the no-choice experiment. The data for food consumption and nutrient intake during the 5th instar stage are illustrated in (**a**,**c**), and the data for food consumption and nutrient intake during the two larval stages are illustrated in (**b**,**d**).

Caterpillar nutrient regulation was explored when they were given no choice in their protein/carbohydrate dietary combination. The findings are presented in Figure 5a,b, and the details of the full parametric models are given in Table 2. From the obtained results, it was clear that changes in the diet’s nutrient content significantly affected the insect’s food consumption and nutrient intake. 

In the case of the 5th instar insects, food consumption increased notably when nutrients were the most diluted. It was also observed that the diet with the highest proportions of carbohydrates, p7:c14, prompted the highest food consumption among the most diluted diets. However, the lowest food consumption did not occur for the most concentrated diet; rather, it occurred for those with a high proportion of proteins, i.e., p35:c7. This pattern shifted slightly when the total amount of food consumed by both larval stages was considered, with the lowest food consumption occurring for diets containing the highest concentration of nutrients (Figure 5b). The pattern of nutrient intake did not align directly with food consumption. Nutrient intake by the insects tended to increase when the food was more carbohydrate-biased, demonstrating that their intake was more affected by the p/c ratios than by the foods’ nutrient concentration (Figure 5c,d).

#### 3.2.2. Performance across the Nutrient Landscape

**Figure 6:**
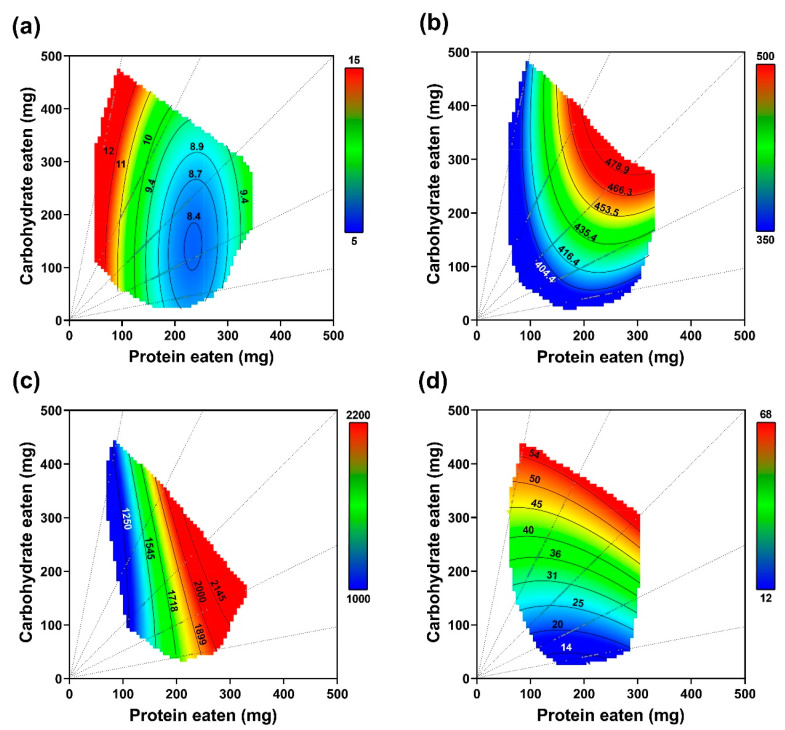
Thin-plate spline visualizations of the response surface for developmental time (**a**), pupal mass (**b**), fecundity (**c**), and lipid accumulation (**d**) on the eleven diets in the no-choice treatment.

The physiological consequences represented by developmental time, pupal mass, fecundity, and lipid accumulation due to different diets are presented in Figure 6. The statistical significance for the models indicated that changes in these independent variables correlated with the nutrient content in the diets (Table 3).

No significant correlation was found between dietary carbohydrate content and developmental time, but developmental time was significantly associated with the intake of proteins. This finding is visually demonstrated in Figure 6a with parallel color patches along the *y*-axis (carbohydrate axis). There was a substantial impact of nutrient concentrations on pupal mass. Pupal mass peaked at a nutrient concentration of 63% (Figure 6b). Furthermore, imbalances in the p/c ratio influenced pupal mass, with imbalances in either direction leading to a decrease in pupal mass. There was no notable correlation between dietary carbohydrate content and fecundity according to the data in Table 3. Interestingly, a high pupal mass did not guarantee high reproduction rates, as illustrated in Figure 6b,c. For instance, at the rail of p14:c7, fecundity could be high while pupal mass was low. When considering the effect of the two nutrients on reproduction, proteins were found to have a stronger impact than carbohydrates, with coefficients of 1054.37 and 175.02, respectively. Both dietary proteins and carbohydrates had an influence on lipid accumulation, as demonstrated in Figure 6d. However, dietary carbohydrates influenced lipid accumulation more than dietary proteins, with coefficients of 139.59 and 7.14, respectively. This finding is visually represented by color patches which were positioned more vertically along the *y*-axis (carbohydrate axis) compared to the *x*-axis (protein axis).

## 4. Discussion

Animal nutrition is a complex and dynamic process that impacts development, reproduction, and survival. Previous studies mostly focused on nutritional regulation using varying p/c ratios at a certain nutrient concentration [13,17,21,24,42,46,47,48,49,50]. However, the concentrations of proteins and carbohydrates in plants is also variable depending on the plant species, plant tissue, or growth stage. Moreover, the trade-off between nutritional regulation and fecundity is less well understood. In this study, we showed, for the first time, how *M. separata* regulates nutrient intake to balance different aspects of performance under different nutrient environments. Both dietary proteins and carbohydrates were found to substantially affect physiological parameters, but their impact was not uniform. Proteins may have a greater effect on fecundity, whereas carbohydrates may have a more pronounced influence on lipid accumulation. In addition, both the ratio and concentration of the nutrients had significant effects on food consumption and insect performance. 

In the choice experiment, *M. separata* did not tightly regulate their nutrient intake although they fed discriminately on the paired foods. This is different from many reported cases including some lepidopteran species, which control their intrinsic total nutrient intake under a balanced diet [23,47,48,51]. However, there were also several cases in which the animals (like plant bugs and mason bees) did not tightly regulated their nutrient intake [52,53]. These contrasting results reflect the divergence of nutritional regulation even among lepidopteran species. 

Insects ingested more nutrients, grew bigger, and produced more eggs with increased nutrient concentrations in food. However, the caterpillars did not try to maximize their nutrient intake when provided with a low nutrient concentration food, which may be attributed to increasing food intake to meet their body needs [54]. To ingest the same amount of nutrients, caterpillars have to feed for a longer period of time on the low concentration foods compared with the higher concentration foods, so the animals may strike a balance between feeding duration and nutrition fulfilment, which can be beneficial for them by reducing their predation risk [55,56,57]. 

In the no-choice experiment, the response surface analyses indicated that developmental time and fecundity may be a function of dietary proteins. In plant tissues, the concentrations of proteins and carbohydrates may change simultaneously and correspondingly. For example, when plants mature and senesce, the nutrient composition usually changes from a protein bias to carbohydrate bias [58]. The strong correlation between lipid accumulation and dietary carbohydrates indicated that ‘excess’ carbohydrates can be utilized when the caterpillars encounter low p/c food. However, the reduced intake of protein nutrients can lead to low fecundity. This is exactly consistent with the characteristics of the migrating population, i.e., high lipid accumulation and low fecundity [59,60]. Therefore, we proposed that the caterpillars may prepare for the life history change from a sedentary population to a migrating population through adjusting the p/c ratio that they ingest.

In summary, this study, for the first time, provided comprehensive information on the nutritional regulatory responses of the caterpillar pest *M. separata*. The insect likely adjusts its physiological processes when encountering various nutritional environments, which is in coordination with its life history traits including protein consumption for body growth, lipid accumulation for marching and migration, etc. [17,47]. Understanding these relationships can shed new light on the biological interactions between plant-feeding insects and their food. This work enriches the existing body of knowledge concerning the nutritional ecology of insects and sets the groundwork for further related research.

## Figures and Tables

**Table 1:** MANCOVA and ANCOVA analyses for proteins and carbohydrates consumed by *M. separata* in the choice experiment. F ratios and *p* values from MANCOVA (Pillai’s trace) and ANCOVA are presented with food pairing as the variables, and the newly molted 5th instar larval mass as the covariate to adjust for size differences between insects.

Variable	P and C Consumed			Protein Consumed			Carbohydrate Consumed		
Effect	df	F	*p*	df	F	*p*	df	F	*p*
5th instar									
Food pairing	8,188	27.79	0.000	4,94	14.61	0.000	4,94	31.36	0.000
Covariate	2,93	0.27	0.767	1,94	0.21	0.648	1,94	0.004	0.951
6th instar									
Food pairing	8,188	27.83	0.000	4,94	39.16	0.000	4,94	23.43	0.000
Covariate	2,93	2.59	0.080	1,94	4.74	0.032	1,94	0.23	0.630

**Table 2:** The full parametric model for the effects of proteins and carbohydrates on consumption. Most terms in the model were significant in interpreting the relationship between the independent variable and the dependent variables. For convenience, the *p*-value of insignificant terms are bolded. The coefficient for each term is enclosed in parentheses.

	5th Instar Larvae		5th and 6th Instar Larvae	
Model Terms	Food Consumption	Nutrient Intake	Food Consumption	Nutrient Intake
Full model	F_6,321_ = 151.57 *p* < 0.001	F_6,321_ = 108.39 *p* < 0.001	F_6,321_ = 387.88 *p* < 0.001	F_6,321_ = 158.82 *p* < 0.001
Initial mass	F_1,321_ = 73.20 *p* < 0.001 (51.22)	F_1,321_ = 78.90 *p* < 0.001 (18.46)	F_1,321_ = 3.59 *p* = 0.059 (43.26)	F_1,321_ = 7.50 *p* = 0.007 (24.34)
Protein (P)	F_1,321_ = 164.45 *p* < 0.001 (−44.85)	F_1,321_ = 7.82 *p* = 0.006 (3.39)	F_1,321_ = 1336.05 *p* < 0.001 (−470.37)	F_1,321_ = 2.03 ***p* = 0.155** (7.14)
Carbohydrate (C)	F_1,321_ = 2.47 ***p* = 0.117** (5.54)	F_1,321_ = 421.96 *p* < 0.001 (25.15)	F_1,321_ = 118.89 *p* < 0.001 (−140.93)	F_1,321_ = 769.62 *p* < 0.001 (139.59)
P^2^	F_1,321_ = 139.43 *p* < 0.001 (62.61)	F_1,321_ = 51.93 *p* < 0.001 (13.27)	F_1,321_ = 305.83 *p* < 0.001 (506.73)	F_1,321_ = 83.21 *p* < 0.001 (102.89)
C^2^	F_1,321_ = 2.51 ***p* = 0.115** (8.45)	F_1,321_ = 0.57 ***p* = 0.452** (1.40)	F_1,321_ = 46.04 *p* < 0.001 (197.86)	F_1,321_ = 7.12 *p* = 0.008 (30.28)
P × C	F_1,321_ = 93.72 *p* < 0.001 (53.51)	F_1,321_ = 17.44 *p* < 0.001 (8.01)	F_1,321_ = 138.79 *p* < 0.001 (355.82)	F_1,321_ = 0.39 ***p* = 0.533** (−7.33)

**Table 3:** The full parametric model for the effects of proteins and carbohydrates on developmental duration, pupal mass, lifetime fecundity, and lipid accumulation. Most terms in the model were significant in interpreting the relationship between the independent variable and the dependent variables. For convenience, the *p*-value of insignificant terms are bolded. The coefficient for each term is enclosed in parentheses.

Model Term	Developmental Time	Pupal Mass	Fecundity	Lipid Accumulation
Full model	F_6,321_ = 61.78 *p* < 0.001	F_6,316_ = 41.84 *p* < 0.001	F_6,48_ = 10.48 *p* < 0.001	F_6,206_ = 256.81 *p* < 0.001
Initial mass	F_1,321_ = 16.18 *p* < 0.001 (−0.96)	F_1,316_ = 32.70 *p* < 0.001 (26.81)	F_1,48_ = 0.48 ***p* = 0.493** (96.05)	F_1, 206_ = 4.52 *p* = 0.035 (1.97)
Protein (P)	F_1,321_ = 97.06 *p* < 0.001 (−2.71)	F_1,316_ = 113.58 *p* < 0.001 (60.52)	F_1,48_ = 43.07 *p* < 0.001 (1054.37)	F_1,206_ = 21.76 *p* < 0.001 (5.20)
Carbohydrate (C)	F_1,321_ = 3.18 ***p* = 0.076** (0.34)	F_1,316_ = 98.33 *p* < 0.001 (38.09)	F_1,48_ = 2.59 ***p* = 0.114** (175.02)	F_1,206_ = 828.32 *p* < 0.001 (21.85)
P^2^	F_1,321_ = 22.64 *p* < 0.001 (3.86)	F_1,316_ = 8.96 *p* = 0.003 (−53.69)	F_1,48_ = 0.62 ***p* = 0.434** (−323.21)	F_1,206_ = 5.72 *p* = 0.018 (8.67)
C^2^	F_1,321_ = 3.07 ***p* = 0.081** (0.52)	F_1,316_ = 3.66 ***p* = 0.057** (−11.68)	F_1,48_ = 0.12 ***p* = 0.734** (64.99)	F_1,206_ = 0.84 ***p* = 0.361** (−1.08)
P × C	F_1,321_ = 0.201 ***p* = 0.654** (−0.33)	F_1,316_ = 7.00 *p* = 0.009 (40.95)	F_1,48_ = 0.21 ***p* = 0.650** (216.24)	F_1,206_ = 4.62 *p* = 0.033 (6.21)

## Data Availability

The data presented in this study are available from the corresponding author upon reasonable request.
